# SHORT TELOMERES ASSOCIATE WITH HYPERPHOSPHORYLATED TAU BURDEN IN PRIMARY AGE-RELATED TAUOPATHY

**DOI:** 10.1093/geroni/igac059.1739

**Published:** 2022-12-20

**Authors:** Corey McMillan, Bandita Adhikari, Kurt Farrell, David Wolk, PART Working Group, Edward Lee, John Crary, F Bradley Johnson

**Affiliations:** University of Pennsylvania, Philadelphia, Pennsylvania, United States; University of Pennsylvania, Philadelphia, Pennsylvania, United States; Icahn School of Medicine at Mount Sinai, New York City, New York, United States; University of Pennsylvania, Philadelphia, Pennsylvania, United States; Icahn School of Medicine at Mount Sinai, New York City, New York, United States; University of Pennsylvania, Philadelphia, Pennsylvania, United States; Icahn School of Medicine at Mount Sinai, New York City, New York, United States; University of Pennsylvania, Philadelphia, Pennsylvania, United States

## Abstract

Aging is a major risk factor for hyperphosphorylated tau burden (p-tau). Telomeres lengths (TL) shorten with age and various sources of DNA damage, thus provide a measure of biological age. Additionally, DNA methylation (DNAm) changes over time and may contribute to changes in TL. We hypothesize that shorter TL will be associated with increased risk of p-tau burden and that this process may be mediated by DNAm. We extracted DNA from frontal cortex of 113 individuals (Age=87.3 + 9.3; 37% Female) that met neuropathological criteria for primary age-related tauopathy (PART), characterized by p-tau in the absence of amyloid pathology. We measured mean TL using qPCR to determine the copy number of telomere repeat DNA in comparison to a single copy gene. We also measured DNA methylation using the Illumina MethylationEPIC Kit for ~850K CpGs. P-tau was measured in medial temporal cortex using an Aperio Digital Pathology Slide Scanner. Linear regression revealed that shorter TL was associated with increased p-tau burden (B=-0.28; p=-.003), including adjustment for age (B=0.003; p=0.003). eWAS identified six CpGs associated with TL (all q< 0.05). Causal mediation analyses identified that two of these CpGs mediate the TL and p-tau association: proportion mediated by cg08701686 (UNC5D) and cg24533059 (near IFNGR1 and OLIG3) was 32.5% and 48.6%, respectively. Shorter TL is associated with increased p-tau pathological burden in PART and may be mediated in part by DNAm at particular loci. These findings support the concept that biological aging, as measured with TL and DNAm, may contribute to tauopathy beyond chronological aging.

